# Beyond the Apnea–Hypopnea Index: Circulating Biomarkers and Device-Based Metrics for Cardiometabolic Risk Stratification in Obstructive Sleep Apnea

**DOI:** 10.3390/jcm15103668

**Published:** 2026-05-10

**Authors:** Dumitru Cătălin Sârbu, Mara Andreea Vultur, Maria Beatrice Ianoși, Hédi-Katalin Sárközi, Dragoș Huțanu, Edith Simona Ianoși

**Affiliations:** 1 Pulmonology Clinic, Mureș County Clinical Hospital, 540011 Târgu Mureș, Romania; sarbu.dcatalin@gmail.com (D.C.S.); ianosi.maria-beatrice@stud18.umfst.ro (M.B.I.); 2 Pulmonology Department, George Emil Palade University of Medicine, Pharmacy, Science and Technology of Târgu Mureș, 540139 Târgu Mures, Romania; mara.vultur@umfst.ro (M.A.V.); hedi.balog@umfst.ro (H.-K.S.); edith.ianosi@umfst.ro (E.S.I.); 3 Doctoral School of Medicine and Pharmacy, George Emil Palade University of Medicine, Pharmacy, Science, and Technology of Târgu Mureș, 540139 Târgu Mureș, Romania

**Keywords:** obstructive sleep apnea, apnea–hypopnea index, biomarker, cardiovascular risk, metabolic risk

## Abstract

Obstructive sleep apnea (OSA) is increasingly recognized not merely as a localized anatomical airway disorder, but as a complex systemic condition. While the apnea–hypopnea index (AHI) remains the traditional standard for diagnosis, it possesses inherent limitations in adequately predicting downstream adverse outcomes, necessitating the adoption of novel, comprehensive markers for refined risk stratification. This narrative review summarizes recent evidence on circulating biomarkers and device-based metrics that may complement the AHI for risk stratification in OSA. Beyond traditional metrics, emerging biomarkers in obstructive sleep apnea offer a multifaceted view of the disease, utilizing inflammatory, vascular, metabolic and novel molecular indicators to enhance clinical characterization. These biomarkers reflect key pathophysiological mechanisms, including systemic inflammation, endothelial dysfunction, metabolic imbalance and altered gut microbiota. Commonly studied markers show associations with disease severity and adverse health outcomes. In addition, novel molecular markers, such as microRNAs and advances in sleep study metrics, provide further insight into disease burden, prognosis and cardiovascular comorbidities. A multidimensional framework integrating accessible laboratory markers with device-based metrics may improve identification of patients with OSA who are at highest risk for cardiovascular and metabolic complications.

## 1. Introduction

Obstructive sleep apnea (OSA) is one of the most common sleep-related breathing disorders, characterized by recurrent upper airway collapse during sleep leading to intermittent hypoxia, autonomic fluctuations, sleep fragmentation, and excessive daytime sleepiness [1,2]. Current estimates suggest that nearly 1 billion adults worldwide may be affected, underscoring its substantial public health impact [2,3,4]. Beyond its high prevalence, OSA is strongly associated with cardiovascular and metabolic comorbidities, including hypertension, coronary artery disease, heart failure, stroke, arrhythmias, and increased mortality, as well as significant neurocognitive and socio-economic burden [1,2,4,5,6]. Despite this, OSA remains markedly underdiagnosed across many populations, particularly in underserved and high-risk groups, where limited access to diagnostic resources and low symptom recognition hinders timely identification and treatment [1,5,7,8].

Overnight polysomnography (PSG) remains the diagnostic gold standard; however, its use is constrained by high cost, limited availability, and procedural complexity, contributing to delays in care and inequities in access [3,7,9,10]. The apnea–hypopnea index (AHI), although well established, does not predict cardiovascular and metabolic outcomes, prompting growing interest in complementary biomarkers and physiologic indices that better reflect disease heterogeneity and prognostic risk [3,11,12,13]. In this context, classical serum-derived inflammatory and hematologic markers, composite lipid indices, pro-inflammatory cytokines and metabolic biomarkers such as adipokines, indices of insulin resistance (IR), oxidative stress markers, and vitamin D have been explored as candidate tools for early detection, risk stratification, and treatment monitoring in OSA [4,5,6,9,14,15,16]. Parallel advances in device-based and signal-derived metrics, including hypoxic burden, pulse wave amplitude drops (PWADs), sleep apnea-specific hypoxic burden, and digital oximetry biomarkers, have demonstrated added value for characterizing pathophysiologic burden and predicting cardiovascular outcomes beyond the AHI [3,11,12,13,17]. In addition, emerging molecular, metabolomic, and microbiome-based biomarkers may further enable the identification of high-risk endotypes and support precision medicine approaches in the future [13,16,18,19].

This narrative review synthesizes current evidence on classical and novel serum biomarkers and device-based metrics in OSA. Specifically, it addresses three key questions: (1) How classical serum and metabolic biomarkers contribute to predicting OSA presence and severity and their suitability for early detection; (2) To what extent circulating and physiological biomarkers improve estimation of cardiovascular and metabolic burden beyond the AHI; and (3) What future directions and implementation challenges exist for emerging biomarker-guided strategies in routine clinical practice. By integrating these domains, the review aims to provide an evidence-based framework for incorporating novel biomarkers and diagnostic tools into contemporary OSA care pathways and advancing precision medicine in this highly prevalent disorder [2,3,11,12,13].

Studies were identified by searching MEDLINE/PubMed, Web of Science, Scopus and Cochrane databases using the keywords “obstructive sleep apnea”, “biomarker”, “cardiovascular risk” and “metabolic risk” for the publication years from 2019 to 2025. Additional targeted searches were conducted for specific biomarkers and device-based metrics. Eligible studies included human research articles published in English that examined biomarkers or device-derived metrics associated with OSA and related cardiometabolic risk. In some cases, older studies were included when they provided foundational evidence, landmark findings or unique methodological insights that were not superseded by more recent work. Studies were selected for conceptual relevance and interpretive value rather than through a formal systematic review workflow. Consequently, this approach introduces an inherent risk of selection bias and may not provide exhaustive coverage of all available evidence. Although this narrative review cites specific statistical correlations (e.g., coefficient values) to illustrate potential biological relationships, these estimates often arise from heterogeneous observational studies and therefore should be interpreted cautiously, given their generally weak to moderate strength and the influence of varying sample sizes, study designs, and potential unadjusted confounders.

## 2. Circulating Biomarkers

### 2.1. Inflammatory and Immune Dysregulation

Systemic inflammation is considered a key pathway linking obstructive sleep apnea with its cardiometabolic and neuroinflammatory complications. Recent studies have demonstrated that systemic inflammation, as measured by indices derived from the complete blood count, is closely associated with the severity of obstructive sleep apnea. In a cross-sectional analysis of 263 patients, elevated levels of the systemic immune-inflammation index (SII), systemic inflammation response index (SIRI), neutrophil-to-lymphocyte ratio (NLR) and pan-immune inflammation value (PIV) were observed in those with severe OSA compared to those with a milder form of the condition [20]. Further supporting these findings, Shahul et al. further reported strong discriminatory performance of SIRI for severe OSA (AUC 0.960, cut-off: 1.105; sensitivity: 92.2%; specificity: 91.4%) and accuracy improved further when SIRI was combined with STOP-BANG (AUC 0.983, cut-off: 4; sensitivity: 96.6%; specificity: 100%) [21].

Across the broader literature, NLR is also increased in OSA and is associated with disease severity, increased cardiovascular risk and all-cause mortality, particularly among individuals with coronary heart disease [20,22,23]. These results highlight the potential utility of routine hematological indices, particularly NLR, SIRI and SII, as accessible and cost-effective biomarkers for identifying patients at risk for severe OSA, guiding early intervention strategies with minimal cost, thus preventing major cardiovascular events. Accordingly, these markers should currently be viewed as pragmatic risk-enrichment tools rather than stand-alone prognostic biomarkers.

On the other hand, acute-phase proteins and inflammatory cytokines such as C-reactive protein (CRP), interleukin-6 (IL-6), tumor necrosis factor-α (TNF-α), interleukin-8 (IL-8) and S100 calcium-binding protein B (S100B) have attracted growing interest [24]. In a case–control study that analyzes both the serum and plasma concentrations of these biomarkers, patients with OSA showed roughly double the concentrations of CRP and IL-6 in serum and 1.3–1.7-fold higher TNF-α and IL-8 in plasma compared with controls [25]. These markers increased with OSA severity, older age and higher BMI, but their ability to discriminate OSA from non-OSA was only moderate, suggesting that they may complement rather than replace AHI-based assessment [25].

CRP, one of the most accessible markers in this group, has also been prospectively linked to incident OSA in four U.S. cohorts, with particularly strong associations in younger adults, individuals with lower BMI, and premenopausal women [26]. Likewise, severe OSA has been independently associated with CRP levels above 3 mg/L (adjusted OR: 1.70, 95% CI 1.18 to 2.44) and this relationship is further strengthened in the presence of obesity [27]. However, its specificity is limited because its levels are strongly confounded by obesity, age, sex, comorbidities and acute inflammation and it may not fully discriminate OSA-related risk from other inflammatory states [28,29].

Plasma S100B, a marker of neuronal and glial injury, was also elevated in OSA and supports the concept of concomitant systemic and neuroinflammatory stress [25]. Despite strong biological plausibility, these markers are best regarded as mechanistically informative rather than routine tools for individualized clinical decision-making.

### 2.2. Vascular Injury and Remodeling

Vascular injury and endothelial remodeling represent another major pathway through which OSA may contribute to atherosclerosis and cardiometabolic disease. Biomarkers in this domain can be broadly grouped into endothelial adhesion/dysfunction markers, structural remodeling proteins and oxidative stress indicators [30]. Overall, the available literature supports an association between these pathways and OSA severity, but most data remain observational and heterogeneous [30,31,32,33,34].

Adhesion molecule levels, specifically Vascular Cell Adhesion Molecule-1 (VCAM-1) and Intercellular Adhesion Molecule-1 (ICAM-1), promote leukocyte migration into the vascular wall and endothelial cell proliferation, driving vascular inflammation and atherosclerosis; VCAM-1 appears particularly critical for initiating atherosclerotic lesions, with ICAM-1 providing additional pro-inflammatory support [35,36]. In OSA, both VCAM-1 and ICAM-1 are generally elevated and correlate positively with AHI, with the highest concentrations observed in severe disease [30,31,32,33,34]. In addition, elevated ICAM-1 levels are independently associated with an increased 8-year risk of cardiovascular events (HR 3.65, 95% CI 1.40 to 9.53, *p* = 0.00) in a retrospective cohort study, highlighting its potential as a CVD risk biomarker in this population [30,31,32,33,34].

Matrix metalloproteinase-9 (MMP-9) is a zinc-dependent endopeptidase upregulated by intermittent hypoxia and inflammation in obstructive sleep apnea, promoting vascular remodeling and endothelial dysfunction by degrading extracellular matrix in vulnerable atherosclerotic plaques [37,38]. Consequently, MMP-9 serves as a potential mediator linking OSA severity to a greater cardiovascular and metabolic risks, leading to arterial hypertension, coronary artery disease, stroke and type 2 diabetes [37,38,39,40]. Moreover, M. Li et al. in a case–control study reported that elevated MMP-9 was associated with mild cognitive impairment due to vascular damage and blood–brain barrier breakdown in OSA patients, potentially reflecting neurovascular injury and blood–brain barrier dysfunction [41]. Although there are promising links between MMP-9 and the cardiovascular burden in OSA patients, studies remain limited by number, sample sizes and methodological heterogeneity to validate its clinical use.

### 2.3. Metabolic Dysfunction and Adiposity-Related Markers

Over the past years, there has been an increasing interest in lipid-derived markers, insulin resistance surrogates and adiposity-related mediators as candidate biomarkers linking OSA with metabolic dysfunction. This domain is particularly relevant because dyslipidemia, visceral adiposity and insulin resistance are highly prevalent in OSA patients and may both mediate and confound observed associations. As a result, these biomarkers are clinically attractive, inexpensive and broadly available.

In patients with OSA, triglyceride (TG) levels are significantly higher compared to controls and increase with disease severity, even in non-obese and non-diabetic individuals [42,43]. This pattern is biologically plausible, as intermittent hypoxia can inhibit lipoprotein lipase activity and increases hepatic lipid production, thereby contributing to atherosclerosis and elevated cardiovascular risk [42,44]. Popadic V. et al. in a recent retrospective observational cohort study states that triglyceride levels ≥ 1.7 mmol/L are significantly associated with severe OSA (OR: 1.961, 95% CI 1.251 to 3.072, *p* = 0.003), underscoring the contribution of hypertriglyceridemia to OSA-related vascular burden [42]. Additionally, a population-based study reported that elevated triglycerides are associated with a 32–56% higher likelihood of having OSA or pre-OSA [45].

Triglyceride-based indices are increasingly studied as biomarkers of insulin resistance (IR) and cardiometabolic risk. Recent work evaluates them as promising screening tools for OSA and as prognostic markers within OSA cohorts. The triglyceride–glucose index (TyG) is a robust surrogate marker of IR and has also shown value for identifying OSA risk (AUC 0.681, 95% CI 0.627 to 0.735, *p* < 0.0001, I2 = 80.4%) [46,47,48]. Multiple studies and meta-analyses demonstrate that the TyG index is significantly higher in patients with OSA than in controls, correlates with disease severity and is independently associated with disease progression, particularly in non-obese and hypertensive populations [48,49,50]. The TyG index was found to outperform HOMA-IR in predicting lifetime cardiovascular risk in OSA groups (OR of 4.32, 95% CI 1.19 to 15.67) and is associated with adverse outcomes, including cardiovascular diseases and all-cause mortality [51,52]. Notably, the triglyceride-to-HDL cholesterol (TG/HDL-C) was studied in the NHANES (National Health and Nutrition Examination Survey) cohort and it demonstrated superiority in predicting OSA-associated cardiovascular risk compared to non-HDL-C or TG measurements [53].

TyG composite indices such as TyG–Body Mass Index (TyG-BMI), TyG–Waist Circumference (TyG-WC) and TyG–Waist-to-Height Ratio (TyG-WHtR) were studied in population-based NHANES analyses and they outperformed TyG alone in predictive accuracy of OSA [54]. Particularly TyG-WC was found to be the strongest predictor in both all-cause and cardiovascular mortality risk [54,55]. Moreover, a meta-analysis involving ten studies with a total of 16,726 patients, revealed that the TyG-WC index predicted myocardial infarction risk in hypertensive OSA groups, supporting implementation in routine practice and patient clustering [48]. Integrating these lipid biomarkers and compound indices into risk stratification protocols may improve the identification of OSA patients who are most vulnerable to cardiovascular and metabolic complications.

High-Density Lipoprotein Cholesterol (HDL-C) is traditionally regarded as a protective lipoprotein due to its antioxidant activity [42]. In patients with OSA, HDL-C levels are generally reduced and show an inverse relationship with AHI, reflecting elevated systemic inflammation and consequently an increased risk of cardiovascular morbidity [43,44,56]. By contrast, Non-High-Density Lipoprotein Cholesterol (non-HDL-C) is a calculated value (total cholesterol–HDL-C) and it reflects the total burden of all atherogenic particles containing apolipoprotein B and triglyceride-rich remnants [43,44]. Non-HDL-C levels show a significant positive correlation with OSA severity and hypoxia parameters, and thus they are considered a superior marker for residual atherosclerotic risk than LDL alone [43,44].

Apolipoprotein A-I and B (apoA-I, apoB), protein components of lipoproteins that transport cholesterol and triglycerides, are gaining attention as integrative markers of metabolic risk and atherosclerotic disease. ApoB may offer a more direct measure of atherogenic particle burden than LDL cholesterol, particularly in diabetes, obesity and hypertriglyceridemia, conditions highly prevalent in OSA patients [57]. On the other hand, Fadaei et al. in a case–control study including 92 subjects found that apoA-I significantly decreased in the OSA group, but was stable between OSA categories, indicating a higher atherosclerotic disease setting, supporting the concept of impaired anti-atherogenic capacity [58]. Ultimately, a 4010-patient large-scale observational, cross-sectional study conducted within the Shanghai Sleep Health Study cohort states that the apoB/apoA-I ratio is an independent predictor of IR and is associated with higher predicted 10-year CVD risk (Framingham CVD risk score), reinforcing its potential relevance [59,60].

Another candidate biomarker for metabolic dysfunction associated with OSA is 25(OH)D (vitamin D); lower serum concentrations promote pro-inflammatory cytokines (interleukin-2, interferon-γ, TNF-α) and reduce anti-inflammatory mediators, potentially amplifying OSA-related systemic and upper-airway inflammation and pharyngeal muscle strength deficiency [61,62,63]. In a small observational cross-sectional study, BMI and serum 25(OH)D levels, but not their interaction, served as the primary predictors of insulin resistance in OSA patients [64]. Vitamin D deficiency may therefore be better interpreted as a modifier of metabolic vulnerability in OSA than as a specific biomarker of disease severity.

Adipokines, particularly leptin and adiponectin, are bioactive proteins primarily secreted by adipose tissue that play critical roles in regulating appetite, lipid metabolism and inflammation [65,66]. In the pathophysiology of obstructive sleep apnea, these molecules are deeply involved through mechanisms linked to intermittent hypoxia and sleep fragmentation, which induce oxidative stress and systemic inflammation [66]. Key results from studies indicate that leptin levels are frequently elevated in OSA patients compared to controls, suggesting a state of leptin resistance that correlates positively with disease severity indices, specifically AHI [66,67]. Conversely, adiponectin, which possesses anti-inflammatory and insulin-sensitizing properties, is often found to be decreased in OSA patients, although some research has reported elevated levels or no significant variation across different OSA phenotypes [66,67,68,69,70]. An observational cross-sectional study found that patients with OSA had significantly lower serum adiponectin levels than healthy controls, showing diagnostic potential at a serum cut-off of 5 mg/L (AUC 0.837, sensitivity: 78%, specificity: 89%) [71]. Despite this signal, adipokines remain influenced by obesity and other metabolic comorbidities and their incremental value beyond routine metabolic assessment still requires clarification.

**Figure 1:**
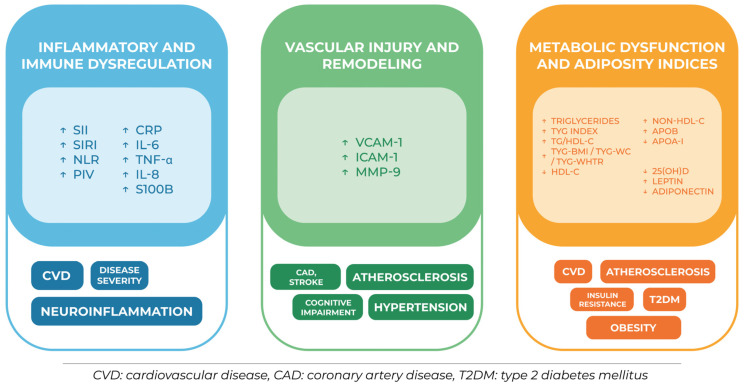
Circulating biomarkers and their prognostic role for cardiometabolic diseases. ↑: increased levels, higher; ↓: decreased levels, lower.

Taken together, these circulating biomarkers reflect key mechanisms linking OSA to cardiometabolic diseases. Despite the limited clinical validation of specific cut-off values for most markers, their integrative use may substantially improve risk assessment and prognosis, as summarized in Figure 1.

## 3. Exploratory Biomarkers

### 3.1. The Sphingolipid Rheostat

The sphingolipid rheostat, defined by the dynamic balance between pro-apoptotic ceramides and pro-survival sphingosine-1-phosphate (S1P), appears critically dysregulated in OSA, serving as a key molecular mechanism linking the sleep disorder to cardiovascular comorbidities such as arterial hypertension and atherosclerosis [72,73]. Intermittent hypoxia and pro-inflammatory cytokines drive the production of ceramides, which impair endothelial function by uncoupling nitric oxide synthase and promoting oxidative stress, thereby increasing cardiovascular vulnerability [73,74]. S1P usually supports endothelial barrier function and vascular tone, yet in OSA its protective role may be attenuated or context dependent: reduced levels may leave the endothelium more susceptible to ceramide-mediated injury, whereas excessive signaling may also have maladaptive endocrine and inflammatory effects [72]. The shift in this rheostat toward ceramide accumulation or aberrant S1P signaling thus underpins the vascular inflammation and metabolic dysfunction observed in sleep apnea patients [72,73,74]. Although biologically compelling, this pathway is still at an early translational stage and is not ready for routine clinical use.

### 3.2. Microbiome-Associated Biomarkers

The gut microbiota forms a highly dynamic microbial community that helps maintain intestinal barrier integrity and overall homeostasis; when this ecosystem is disturbed, barrier permeability can increase, driving systemic inflammation and cardiometabolic disease processes that are now increasingly linked to obstructive sleep apnea [75]. Li et al., in a 2023 observational case–control study including 48 patients, identified a robust association between OSA severity, impaired intestinal barrier integrity and targeted shifts in the gut microbiome [76]. Compared with controls, OSA patients showed significantly higher plasma levels of the barrier dysfunction biomarkers, D-lactic acid (D-LA) and intestinal fatty acid–binding protein (I-FABP), indicating increased intestinal permeability and epithelial injury [76,77,78]. Consistent with this, beta-diversity analyses demonstrated clear differences in microbial community structure not only between severe OSA and non-OSA patients, but also between OSA severity groups, supporting the idea that barrier damage tracks with a distinct dysbiosis profile [76,79]. Specifically, elevated D-LA levels were positively correlated with an enrichment of the genera *Fusobacterium*, *Megamonas* and *Lachnoclostridium*, while high I-FABP levels were associated with enriched *Alloprevotella* [76]. These findings are clinically meaningful, given that *Fusobacterium nucleatum* has been linked to cardiovascular disease in recent research [80].

Taken together, these results suggest that OSA-related gut barrier disruption and accompanying microbial shifts may contribute to systemic inflammation and may help explain the elevated cardiovascular comorbidity burden observed in OSA [76,77,78,79,80]. Although this is an intriguing and biologically coherent signal, it remains exploratory: sample sizes are modest, methodologies vary and microbiome signatures are not yet standardized for clinical use.

### 3.3. MicroRNAs

Molecular profiling of microRNAs has revealed their diagnostic and prognostic importance, positioning them as promising regulators and biomarkers in cardiovascular diseases. An observational longitudinal study conducted by Santamaria-Martos F. that included 230 patients was able to validate in the men cluster six downregulated microRNAs (miRNA) in the OSA group compared to the non-OSA group (miR-133a, miR-181a, miR-199b, miR-340, miR-345 and miR-486-3p) [81]. These microRNA particles are involved in pathways related to risk factors and diseases commonly associated with OSA: miR-133a regulates cardiomyocyte proliferation and is dysregulated in myocardial infarction, miR-199b suppression predicts unfavorable outcomes for colorectal cancers, miR-486-3p is associated with insulin and glucose metabolism, miR-340 is associated with pathways related to melanoma, miR-345 is correlated with the AHI and showed statistically significant recovery after CPAP treatment and miR-181a downregulation is associated with atherosclerosis vascular inflammation [81,82,83,84,85,86,87,88]. This downregulation is likely due to chronic hypoxia impairing the cellular machinery (Dicer) required to produce miRNAs [89]. Furthermore, combining molecular biomarkers (miRNAs) with clinical questionnaires, such as NoSAS score, improve diagnostic precision by achieving the highest accuracy [81].

MicroRNA-107 (miR-107) plays a pivotal role in regulating key metabolic and signaling pathways; specifically, it controls glutamate metabolism, serotonin synthesis and signaling and adiponectin expression [71]. The literature data describes the downregulation of miR-107 as a pathway to hypertension in OSA patients, being identified to contribute to vascular remodeling in hypoxic conditions [90]. Furthermore, in a small cross-sectional study assessing its diagnostic value, miR-107 showed its ability to distinguish cases from controls at a serum cut-off of 3.0 ng/mL (AUC 0.974, sensitivity: 100%, specificity: 89.3%) [71]. However, given the limited sample size, these findings should be interpreted as preliminary; more data is needed to validate the presumed cut-off values and its characteristics.

Nevertheless, these findings should currently be regarded as exploratory. Assay standardization, reproducibility across cohorts, sex-specific effects and external validation remain major barriers before microRNAs can be positioned as clinically actionable biomarkers.

## 4. Device-Based Metrics

### 4.1. Sleep Apnea-Specific Hypoxic Burden and Surrogates

Recent work strongly supports sleep apnea-specific hypoxic burden (HB/SASHB) as a robust marker and has some of the strongest evidence for cardiovascular and, more recently, metabolic risk stratification beyond the AHI [91,92]. Evidence now spans large community cohorts and randomized trial reanalysis. HB/SASHB quantifies the area under the desaturation curve for each respiratory event (frequency × depth × duration), normalized by sleep time, and is explicitly designed to be specific to OSA. Among the currently available device-based metrics, hypoxic burden appears relatively mature from a translational perspective, although universal cut-offs and implementation pathways are still lacking. However, several studies have used empirically derived thresholds to stratify risk in specific populations. A study conducted within the Pays de la Loire Sleep Cohort, including 5358 patients with OSA without overt cardiovascular disease at the time of the diagnostic sleep study, found that hypoxic burden could be a strong predictor of major adverse cardiovascular events, particularly among women and younger patients [93]. To further support these findings, Azarbarzin et al. also showed, across two major cohorts, that SASHB correlates with a higher prevalence of heart failure (HF) in men and predicts HF more effectively than AHI, suggesting its potential value as a marker of mortality [94]. Furthermore, a cross-sectional study including 2173 patients with suspected OSA demonstrated that SASHB is independently associated with multiple markers of abnormal glucose and lipid metabolism and may serve as a valuable predictive metric for glycolipid metabolic disorders in patients with OSA [91].

Oximetry-derived hypoxic burden (HB_Oxi_) is a metric defined as the total area under all automatically identified oxygen desaturation (based on a >2% desaturation threshold) curves divided by total sleep time using a pulse oximeter [95]. HB_Oxi_ was highly correlated with manually scored HB (Spearman 0.81, *p* < 0.001) and showed similar associations with excessive daytime sleepiness, hypertension and CVD mortality supporting scalability, highlighting its future greater accessibility [95].

### 4.2. Pulse Wave Amplitude Drop

A pulse wave amplitude drop (PWAD) is defined as a sudden reduction of more than 30% in pulse wave amplitude (PWA), lasting several heartbeats and followed by recovery, reflecting transient vasoconstriction in the finger microvasculature driven by sympathetic activation and changes in vascular tone, as measured by the photoplethysmography signal, which captures pulsatile blood volume changes in the fingertip with each heartbeat. In obstructive sleep apnea patients, respiratory events and arousals repeatedly trigger autonomic activations and pulse wave amplitude drops. Lower PWAD index (fewer drops per hour), longer PWAD duration and greater area under the curve are consistently linked to increased odds of hypertension, diabetes and prior cardiovascular events in large population studies (HypnoLaus general population cohort) [96,97]. In sleep clinic cohorts, a lower PWAD index and longer duration are also associated with established or elevated Framingham 10-year cardiovascular risk scores, suggesting that PWAD features provide complementary information to traditional risk stratification in patients with and without obstructive sleep apnea [98]. Furthermore, some authors propose mechanistic pathways linking OSA and cardiovascular risk through PWAD properties, whereby OSA-related intermittent hypoxia, sleep fragmentation and intrathoracic pressure swings may blunt baroreflex sensitivity and promote endothelial dysfunction, arterial stiffness and chronic sympathetic overactivity, limiting normal vasoconstriction–vasodilation responses, reducing the PWAD index and increasing cardiovascular risk [9]. This biomarker may represent a practical tool for cardiovascular risk stratification both in OSA patients and in the general population, as its assessment does not require full polysomnography recording but can be derived from simple overnight pulse oximetry recording.

### 4.3. Heart Rate Response and Heart Rate Variability

OSA induces cyclical fluctuations in heart rate due to repeated apneas/hypopneas followed by arousals and reoxygenation. These cycles reflect acute vagal activation during apnea (bradycardia), followed by sympathetic surges upon event termination (tachycardia) [17]. Two distinct metrics were used in research to characterize the heart rate response: ΔHR, the manually scored difference between the peak heart rate and the preceding minimum in relation to a given event, and ΔHR_oxi_, in which the difference is specifically referenced to oxygen desaturation event, automatically scored from a single-channel pulse oximetry [17,99]. A study on two distinct European cohorts has shown that these metrics are strongly correlated with each other and showed the same level of association with incident cardiovascular events [99]. Moreover, the ΔHR_oxi_ outperformed the manually scored ΔHR by demonstrating stronger effect sizes for predicting major adverse cardiovascular events and by showing significant associations with a broader range of specific adverse outcomes, including all non-fatal cardiovascular events, stroke and all-cause mortality [99]. However, several limitations persist, including the limited ability of pulse oximetry-derived pulse rate to capture autonomic regulation versus ECG-based HRV (with arrhythmia-related confounding), heterogeneity in ΔHR analytic thresholds and underexplored effects of comorbidities. Despite these challenges, ΔHR_oxi_, underscores the need for automated, wearable-integrated measurement tools that can support continuous monitoring of treatment adherence and long-term cardiovascular risk in patients with OSA alongside other metrics.

Heart rate variability (HRV), a non-invasive marker of autonomic nervous system activity, has emerged as a valuable tool for assessing the impact of OSA on cardiovascular integrity and autonomic regulation [100,101]. Multiple studies confirm that OSA is associated with reduced global HRV, particularly lower time-domain indices such as SDNN (standard deviation of NN intervals) and RMSSD (root mean square of successive differences) and altered frequency-domain measures indicating increased sympathetic dominance (higher LF/HF ratio) [100,102,103]. These changes are more pronounced with increasing OSA severity, during both sleep and wakefulness [100,101,102]. The pathophysiology involves intermittent hypoxia, arousals from sleep, oxidative stress, inflammation and endothelial dysfunction, all contributing to sympathetic overactivity and parasympathetic withdrawal [101,103]. Sleep fragmentation further exacerbates sympathovagal imbalance, while comorbid conditions like depression can independently worsen HRV profiles [101,104]. However, there is considerable heterogeneity across studies regarding optimal measurement protocols (e.g., night vs. day recordings), analytic methods (linear vs. nonlinear indices), influence of comorbidities (depression, obesity, diabetes, etc.), age/gender effects and the predictive value of specific HRV parameters for clinical outcomes such as stroke or cognitive decline. Accordingly, HRV is best viewed as a physiologically informative marker of autonomic dysregulation rather than a standardized prognostic tool in current OSA practice.

### 4.4. Sleep Breathing Impairment Index

SBII is a polysomnography-derived index that quantifies how much sleep breathing is impaired by obstructive sleep apnea, combining both how often events occur and how damaging they are, defined as Σ(desaturation area × event duration)/total sleep time [105]. SBII quantifies the total physiological burden of those events, so it is a more comprehensive measure of OSA severity than AHI alone [105,106]. Higher SBII is independently associated with increased risk of major adverse cardiovascular events, atrial fibrillation, heart failure, coronary artery disease, stroke and all-cause mortality, even after adjusting for confounders such as age, BMI, comorbidities and other sleep metrics [105,107,108,109]. In head-to-head analyses within large datasets (Sleep Heart Health Study), SBII demonstrates higher hazard ratios for CVD mortality (HR 2.04, CI: 1.25, 3.34) than AHI or ODI; other novel indices like hypoxic burden also outperform AHI but may be less robust than SBII for certain endpoints [107]. Furthermore, SBII may predict incident diabetes and metabolic syndrome more accurately than AHI, especially when combined with insomnia symptoms (COMISA-SBII phenotype) [109]. Although SBII has demonstrated enhanced predictive value for cardiometabolic risk in recent studies, its clinical applicability remains constrained by methodological complexity, lack of standardization and limited externally validation.

## 5. Sex Differences in OSA Burden

Obstructive sleep apnea differs substantially between women and men in symptoms, physiology and downstream cardiometabolic risk [110,111]. These sex differences intersect with estrogen status, menopause, comorbidity burden and autonomic and inflammatory pathways, with important implications for biomarker selection [111]. For instance, after the menopause, women tend to present symptoms of OSA in correlation to lower serum estrogen and progesterone levels [112]. Conversely, intermittent hypoxia and sleep fragmentation may impair gonadal function and potentially reduce the estrogen levels even in premenopausal women [113]. The decrease in female sex hormones is known to be associated with a greater prevalence of cardiovascular and metabolic diseases, mainly due to the loss of protective anti-inflammatory effects [111,114].

The gender specificity of endothelial dysfunction is not incidental. Estrogen withdrawal contributes to endothelial impairment, while OSA, menopause, and obesity may each independently worsen this risk [115]. Together, these factors can exert additive or even synergistic effects, particularly in postmenopausal women. Moreover, higher arterial stiffness (measured as Augmentation Index, AIx) and higher carotid intima-media thickness was observed in female patients with a greater risk for left ventricular hypertrophy [116].

In relation to arrhythmogenesis, estrogen has been shown to attenuate oxidative stress and decrease reactive oxygen species (ROS) in female cardiomyocytes preventing pathways leading to atrial fibrillation [117].

Postmenopausal women present higher levels of estradiol which is linked to higher levels of CRP and lower adiponectin levels, further creating a setting for inflammation and insulin resistance [118,119].

Regarding differences in cardiovascular and metabolic burden between men and women, there is a strong association between moderate/severe OSA and stage 2 hypertension in men with an increasing prevalence with age, but more women with OSA tend to have elevated arterial blood pressure due to their REM-predominant events and sympathetic activation added to the loss of estrogen protection [120,121]. Men are more prone to developing heart failure with reduced ejection fraction (HFrEF), whereas women more frequently develop HFpEF (heart failure with preserved ejection fraction) with diastolic left ventricular filling more often impaired [113,122,123]. Moreover, a large study stated that in women, OSA is independently associated with elevated high-sensitivity troponin T, left ventricular hypertrophy, and a higher risk of heart failure or death, indicating a stronger link to cardiovascular disease and cardiac remodeling than in men [124].

Women tend to have a greater prevalence of metabolic syndrome, particularly postmenopausal women, with more chances to develop further life-threatening complications without medical intervention [125]. Non-HDL-c and TyG index were found to be positively correlated with AHI in women, but not in male patients [126]. On the other hand, TyG, TyG-BMI and TyG-WC were significantly higher in males [47]. Evidence suggests that in women with OSA, especially younger or non-obese women and those with polycystic ovary syndrome, OSA is associated with a more atherogenic apolipoprotein profile: relatively lower apoA-I, higher apoB and higher apoB/apoA-I ratio [127,128].

Ultimately, OSA is associated with neurocognitive decline in both sexes, but women, especially middle-aged and older women and those with comorbid insomnia or obesity, often show stronger links to memory dysfunction and dementia risk at similar disease severity [129,130,131,132]. Men also exhibit cognitive deficits, particularly with severe, untreated OSA and in older age, but population data tilt toward greater cognitive vulnerability in women [132,133,134].

## 6. CPAP Treatment Effect on Cardiometabolic Risk Biomarkers

Studies from past years consistently show that CPAP treatment can lead to significant reductions in inflammatory and oxidative stress biomarkers, including NLR, PLR, CRP, IL-6, TNF-α and IL-8, along with improvements in oxidative stress markers with promising results as potential predictors of treatment efficacy [4,24,135,136,137]. Conversely, other authors affirm conflicting results after CPAP treatment, reporting similar inflammatory biomarkers values [6,138]. Furthermore, long-term positive airway pressure treatment is associated with higher vitamin D levels, likely via improved overall health and reduced inflammation, but data is mainly observational [62,63,139].

The vascular injury biomarkers tend to decrease after CPAP treatment with lower odds of cardiovascular adverse events, especially ICAM-1 and VCAM-1 [24,34]. When it comes to MMP-9 the results are spare; some studies observed a decrease in MMP-9 levels, but it was not sustained over time in other studies [38,140,141].

Available evidence shows that CPAP can improve standard lipid profile markers (TG, HDL-C, LDL-C) in OSA and some studies also reported improvements in apolipoprotein levels implying a reduced metabolic disease burden [24,142].

Limited data based on small cohorts have shown that mi-RNA profiles can be influenced by CPAP therapy in a positive matter, partially normalizing specific circulating miRNAs and shifting overall omic patterns toward a non-OSA phenotype with a lower inflammatory status [81,143,144]. These findings can create a setting for future precision medicine in terms of assessing the efficacy of CPAP in OSA patients.

SASH/HB, PWAD, and SBII are emerging metrics of cardiovascular risk and appear to identify subgroups that gain more cardiovascular protection from CPAP therapy, especially those with high hypoxic burden [9,99,108,145]. However, current studies provide limited evidence on how CPAP changes these metrics’ values, leaving their role as dynamic markers of treatment response an important area for further research [145,146].

## 7. Clinical Availability and Future Perspectives

Despite the rapidly growing body of evidence linking obstructive sleep apnea to systemic inflammation, vascular injury and metabolic dysregulation, these biomarkers remain primarily confined to the research setting and are available in a heterogeneous manner; see Table 1.

Future research should aim to bridge the gap between exploratory findings and clinically actionable tools, with a particular emphasis on standardized integration into patient-centered care pathways.

A key priority is the prospective validation of candidate biomarkers and physiological metrics in large, well-characterized cohorts with long-term follow-up. Standardized, reproducible cut-off values are essential for improving comparability across studies and supporting clinical implementation.

Another important direction is integrating multimodal data by combining conventional sleep metrics with widely accessible biomarkers and device-derived parameters. These approaches may enable more precise phenotyping of OSA and improve the identification of patients at the highest cardiometabolic risk.

**Figure 2:**
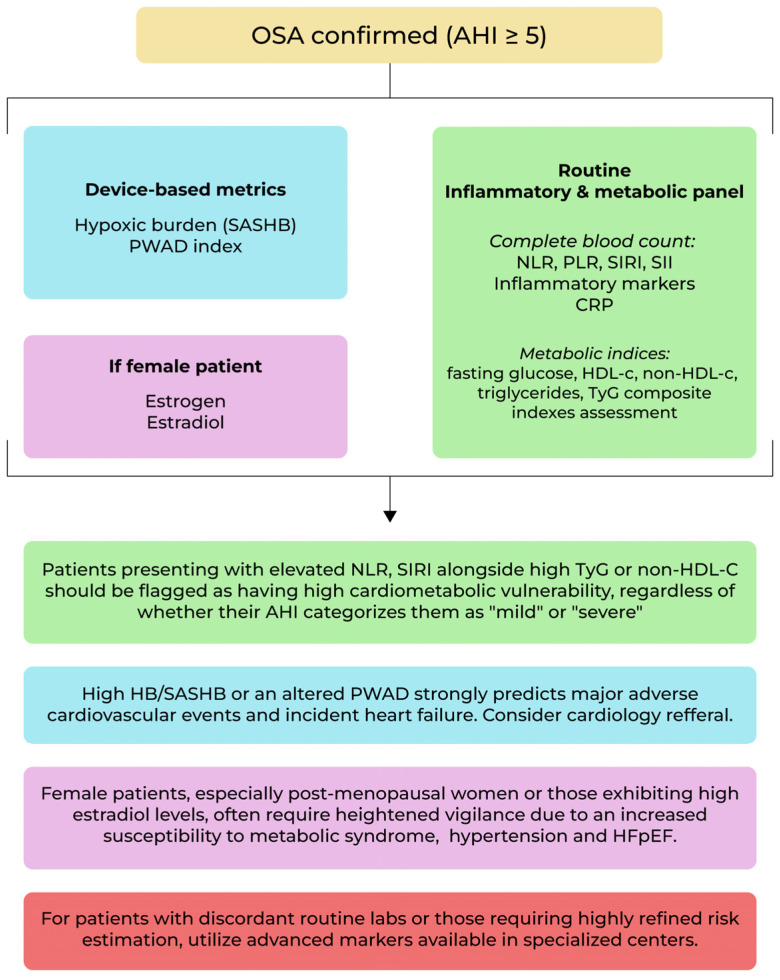
Biomarker-guided assessment for cardiometabolic risk.

Given the available evidence, we propose an algorithm for patients that addresses a sleep study clinic to better assess their cardiovascular and metabolic risk; see Figure 2.

Finally, future studies should evaluate the clinical impact of biomarker-guided risk stratification on therapeutic strategy decisions such as CPAP therapy, weight management and pharmacological treatment.

## 8. Conclusions

Obstructive sleep apnea should be regarded not only as a sleep-related breathing disorder, but also as a complex systemic condition characterized by inflammatory, vascular, metabolic, autonomic and molecular dysregulations. Given their accessibility and low cost, inflammatory blood count indices are useful for assessing OSA severity and systemic inflammation. Lipid profiles and insulin resistance markers provide complementary insights into cardiometabolic risk, while select microRNAs may capture underlying molecular mechanisms including hypoxic stress, endothelial dysfunction, and inflammatory pathways. Device-based physiological metrics further improve upon the apnea–hypopnea index by more comprehensively characterizing respiratory event burden. Collectively, these tools may facilitate identification of high-risk OSA phenotypes and enable more personalized diagnostic and prognostic strategies.

## Figures and Tables

**Table 1:** Reviewed biomarkers, their relevance in OSA and general availability.

Biomarker	Biomarker Class	Mechanistic Relevance in OSA	Availability
SII	Composite CBC-derived inflammation index (neutrophils × platelets/lymphocytes)	Reflects systemic inflammation and immune imbalance. Discriminatory performance for severe OSA but not specific.	Routine
SIRI	Composite CBC-derived inflammation index (neutrophils × monocytes/lymphocytes)	Captures myeloid inflammation. Discriminatory performance for severe OSA but not specific.	Routine
NLR	CBC-derived inflammation index (neutrophils/lymphocytes)	NLR reflects innate–adaptive balance; OSA-induced intermittent hypoxia drives chronic inflammation.	Routine
CRP	Acute-phase systemic inflammation marker	OSA increases CRP via intermittent hypoxia and sympathetic activation.	Routine
Triglycerides	Conventional lipid (energy storage, atherogenic when elevated)	OSA increases TG via IR and sympathetic activation, driving atherogenic dyslipidemia.	Routine
HDL-C	Conventional lipoprotein (anti-atherogenic)	OSA lowers HDL-C and impairs its function, reducing endothelial protection.	Routine
non-HDL-C	Composite atherogenic cholesterol (TC-HDL-C)	Non-HDL-C reflects atherogenic lipoproteins elevated in OSA-related dyslipidemia.	Routine
TyG	Composite insulin resistance index ln[fasting triglycerides (mg/dL) × fasting glucose (mg/dL)/2]	TyG is a robust surrogate linked to CVD and mortality associated with OSA.	Routine
TG/HDL-C ratio	Composite index	TG/HDL ratio reflects atherogenic dyslipidemia and metabolic risk in OSA.	Routine
25(OH)D	Hormone/pleiotropic metabolic modulator	Low vitamin D may worsen inflammation and endothelial dysfunction.	Routine
Adiponectin	Adipokine (insulin-sensitizing, anti-inflammatory)	OSA reduces adiponectin, promoting IR and dyslipidemia.	Routine
HB/SASHB	Integrated hypoxemia metric (%min/h across all events)	Hypoxic burden reflects desaturation severity; predicts HF and CV risk in OSA.	Routine
PWAD	Autonomic/vascular reactivity index from photoplethysmography	PWAD reflects vasoconstriction and autonomic response; low PWAD indicates higher CV risk.	Routine
Estrogen	Sex hormone	Loss of protection (menopause) increases inflammation, OSA severity, and CVD risk.	Routine
IL-6	Pro-inflammatory cytokine	Intermittent hypoxia activates NF-κB and IL-6, linking OSA to IR, endothelial dysfunction and cardiometabolic risk.	Selected Centers
IL-8	Pro-inflammatory chemokine	Promotes neutrophil recruitment; elevated in OSA, especially with cardiovascular comorbidity.	Selected Centers
TNF-α	Pro-inflammatory cytokine	TNF-α inflammation, IR and endothelial dysfunction in OSA.	Selected Centers
apoA-I	Major HDL apolipoprotein (anti-atherogenic)	Low apoA-I indicates impaired HDL function and endothelial dysfunction in OSA.	Selected Centers
apoB	Total atherogenic apolipoprotein	ApoB reflects increased atherogenic particles driven by OSA-related dyslipidemia.	Selected Centers
apoB/apoA-I ratio	Atherogenic/anti-atherogenic balance	High apoB/apoA-I ratio indicates elevated cardiometabolic risk in severe OSA.	Selected Centers
ΔHR	Beat-to-beat autonomic response metric	ΔHR reflects autonomic reactivity to events; higher values indicate greater CV stress.	Selected Centers
ΔHR_oxi_	Surrogate autonomic response metric from pulse oximetry alone	Pulse oximetry-based ΔHR reflects autonomic stress when ECG is unavailable.	Selected Centers
HRV	Autonomic balance metric (sympathetic–parasympathetic)	OSA shifts autonomic balance toward sympathetic dominance, reducing HRV and increasing CV risk.	Selected Centers
SBII	Composite respiratory event + hypoxia index (sum of products of event duration × desaturation area over the night)	SBII integrates event frequency, duration and desaturation severity, capturing total hypoxic burden.	Selected Centers
S100B	Astroglial-derived protein; marker of blood–brain barrier disruption and neural injury	Intermittent hypoxia may cause neural inflammatory injury, increasing circulating S100B.	Research Only
VCAM-1	Endothelial activation/adhesion molecule	OSA induces oxidative stress and endothelial activation; VCAM-1 reflects vascular inflammation.	Research Only
ICAM-1	Endothelial activation/adhesion molecule	Similar to VCAM-1, reflects leukocyte–endothelium interaction driven by OSA-related hypoxia and inflammation.	Research Only
S1P	Bioactive sphingolipid carried mainly on HDL	S1P regulates endothelial and cardiomyocyte function; implicated in OSA-related vascular disease.	Research Only
MMP-9	ECM remodeling/plaque-instability enzyme	Intermittent hypoxia upregulates MMP-9, promoting remodeling and plaque instability.	Research Only
D-lactate	Gut-derived microbial metabolite	Marker of gut permeability; relevance to OSA is theoretical with limited data.	Research Only
I-FABP	Enterocyte injury protein	Indicates enterocyte damage.	Research Only
microRNAs	Regulatory non-coding RNAs	Dysregulated microRNAs link OSA to hypoxia signaling, inflammation and metabolic dysfunction.	Research Only

Abbreviations: SII: Systemic Immune-Inflammation Index, SIRI: Systemic Inflammation Response Index, NLR: Neutrophil-To-Lymphocyte Ratio, CRP: C-Reactive Protein, PIV: Pan-Immune Inflammation Value, IL 6: Interleukin-6, IL 8: Interleukin-8, TNF α: Tumor Necrosis Factor-α, VCAM 1: Vascular Cell Adhesion Molecule 1, ICAM 1: Intercellular Adhesion Molecule 1, S1P: Sphingosine-1-Phosphate, MMP 9: Matrix Metalloproteinase-9, HDL-C: High-Density Lipoprotein Cholesterol, TG: Triglyceride, TyG: Triglyceride-Glucose Index, TG/HDL-C: Triglyceride-To-HDL Cholesterol, apoA-I: Apolipoprotein A-I, apoB: Apolipoprotein B, 25(OH)D: Vitamin D, D-LA: D-Lactic Acid, I FABP: Intestinal Fatty Acid-Binding Protein, HB/SASHB: Sleep Apnea-Specific Hypoxic Burden, PWAD: Pulse Wave Amplitude Drop, ΔHR: Heart Rate Response, HRV: Heart Rate Variability, SBII: Sleep Breathing Impairment Index, OSA: Obstructive Sleep Apnea, AHI: Apnea–Hypopnea Index, IR: Insulin Resistance, BMI: Body Mass Index, CV: Cardiovascular, CVD: Cardiovascular Disease HF: Heart Failure. Availability categories were defined as follows: Routine: Widely available in standard clinical laboratories or sleep studies with established methodologies and broad clinical use. Selected centers: Available in specialized or research-oriented clinical centers, requiring advanced analysis or expertise, with emerging but not fully standardized clinical evidence. Research only: Primarily used in experimental or translational research settings, lacking standardization, validated thresholds, and routine clinical applicability.

## Data Availability

No new data were created or analyzed in this study.

## Abbreviations

The following abbreviations are used in this manuscript:

25(OH)D	Vitamin D
AHI	Apnea–Hypopnea Index
apoA-I	Apolipoprotein A-I
apoB	Apolipoprotein B
BMI	Body Mass Index
COMISA-SBII	Comorbid Insomnia and Sleep Breathing Impairment Index
CPAP	Continuous Positive Airway Pressure
CRP	C-Reactive Protein
CVD	Cardiovascular Disease
D-LA	D-Lactic Acid
HB/SASHB	Sleep Apnea-Specific Hypoxic Burden
HDL-C	High-Density Lipoprotein Cholesterol
HRV	Heart Rate Variability
I-FABP	Intestinal Fatty Acid-Binding Protein
ICAM-1	Intercellular Adhesion Molecule-1
IL-6	Interleukin-6
IL-8	Interleukin-8
IR	Insulin Resistance
MMP-9	Matrix Metalloproteinase-9
NLR	Neutrophil-To-Lymphocyte Ratio
ODI	Oxygen Desaturation Index
OSA	Obstructive Sleep Apnea
PIV	Pan-Immune Inflammation Value
PSG	Polysomnography
PWAD	Pulse Wave Amplitude Drop
RMSSD	Root Mean Square of Successive Differences
S1P	Sphingosine-1-Phosphate
SBII	Sleep Breathing Impairment Index
SDNN	Standard Deviation of NN Intervals
SII	Systemic Immune-Inflammation Index
SIRI	Systemic Inflammation Response Index
TG	Triglyceride
TG/HDL-C	The Triglyceride-To-HDL Cholesterol
TNF-α	Tumor Necrosis Factor-A
TyG	Triglyceride–Glucose Index
TyG-BMI	Tyg–Body Mass Index
TyG-WHtR	Tyg–Waist-to-Height Ratio
TyG-WC	Tyg–Waist Circumference
VCAM-1	Vascular Cell Adhesion Molecule-1
ΔHR	Heart Rate Response
